# Residual imaging abnormalities after acute hamstring strain injury: implications for return-to-sport decision-making

**DOI:** 10.3389/fspor.2026.1924906

**Published:** 2026-09-11

**Authors:** Jincheng Wang, Lunan Zhao

**Affiliations:** School of Sports Science, Qufu Normal University, Qufu, China

**Keywords:** hamstring strain, injury, intramuscular tendon, magnetic resonance imaging, muscle healing, reinjury, return to play, ultrasound

## Abstract

Hamstring strain injuries remain a major cause of time loss and recurrent injury in sprint- and kicking-based sports. MRI and ultrasound are frequently used to confirm diagnosis, characterize anatomical severity, and support prognosis. At the return-to-play (RTP) stage, however, their value is less certain. Persistent imaging abnormalities may reflect incomplete healing, expected remodeling, or potentially relevant structural concerns. Available evidence, derived largely from a limited number of partly overlapping cohorts and predominantly nonoperatively managed non-complete tears, shows that residual edema-like signal, low-signal fibrosis, and persistent intramuscular tendon abnormalities are common at clinical recovery and do not by themselves establish unsafe RTP. Conversely, persistent or new connective tissue gaps and intermuscular edema have shown exploratory associations with reinjury, but these signals arise mainly from small cohorts and have not been independently validated as stand-alone clearance criteria. Evidence for ultrasound at RTP is especially limited; its principal role is targeted, standardized serial assessment of accessible lesions, whereas MRI better characterizes deep and tendon-related anatomy. Imaging should therefore modify rather than dictate RTP decisions and should be integrated with pain response, flexibility, strength, high-speed running exposure, sport-specific performance, previous injury, and psychological readiness. Current evidence does not establish that MRI or ultrasound can reliably distinguish incomplete healing from safe RTP in isolation. Prospective sport-specific studies should test the incremental value of imaging beyond clinical and functional models.

## Introduction

In sports that involve sprinting, acceleration, deceleration, kicking, and high-speed running, hamstring strain injuries are among the most frequent non-contact injuries. In professional football and track and field, these injuries are clinically important not only because they cause time away from sport, but also because reinjury is common soon after return and may result in a longer absence than the index injury (1–3). Sports medicine teams therefore face a deceptively simple question: when is it safe for the athlete to return?

Imaging has become central to the early management of hamstring injury. MRI can characterize the affected muscle, lesion location and extent, cross-sectional area, tendon involvement, and injury severity, while ultrasound is more accessible, dynamic, cost-effective, and repeatable, although more operator dependent (4–7). These features can support diagnostic confirmation and initial prognosis. The more difficult question is whether imaging should influence RTP once the athlete is clinically recovered.

The problem is conceptual as much as technical. Tissue healing and functional readiness are not equivalent. An athlete without clinical symptoms may still exhibit edema, scarring, tendon thickening, or residual gaps on MRI. Conversely, a normal or near-normal scan does not establish preparedness for intense sprinting, fatigue resistance, contact exposure, or sport-specific decision-making. The relevant question is therefore not whether imaging detects residual abnormalities, but whether a particular imaging phenotype adds clinically meaningful information about incomplete healing or reinjury risk.

This Mini Review focuses on persistent MRI and ultrasound findings near RTP after acute hamstring strain injury and considers imaging as a selective decision modifier within a multidimensional RTP framework rather than as an independent clearance test. The evidence discussed is derived mainly from nonoperatively managed injuries; complete proximal tendon avulsions and surgically treated injuries are underrepresented and should not be assumed to follow the same evidence base.

## Literature search and review approach

This narrative Mini Review was informed by a focused PubMed/MEDLINE search from database inception to 16 August 2026 using combinations of ‘hamstring’, ‘muscle injury’, ‘MRI’, ‘magnetic resonance imaging’, ‘ultrasound’ or ‘ultrasonography’, ‘return to play’ or ‘return to sport’, ‘reinjury’ or ‘recurrence’, ‘intramuscular tendon’, and ‘healing’, supplemented by backward citation checking of key reviews and primary studies. We prioritized studies reporting imaging at or near RTP, reinjury outcomes, or the incremental prognostic value of imaging, together with consensus statements and systematic or scoping reviews relevant to RTP criteria. Because this was a narrative review, no formal risk-of-bias grading or quantitative synthesis was performed. Where multiple publications arose from the same or overlapping cohorts, they were treated as non-independent reports rather than independent replications.

## Biological healing versus functional recovery

Skeletal muscle repair after strain injury includes overlapping phases of destruction, repair, and remodeling. Histologically, initial myofiber damage and hematoma are succeeded by inflammation clearance, regeneration of myofibers, capillary growth, and formation of connective tissue scars; subsequent remodeling involves scar maturation and reorganization of the myoconnective unit (8, 9). The stages do not align precisely with clinical milestones. Pain might resolve before the scar has completely matured, and sport-specific abilities could recover while imaging still shows some abnormalities.

Recent MRI-based work provides a useful but explicitly proposed framework for interpreting muscle healing. Isern-Kebschull et al. analyzed 310 MRI studies in 128 athletes selected from a single professional-club database, focusing on injuries requiring more than 40 days to RTP and excluding surgically treated and low-grade cases (10). They proposed MRI phases of destruction, repair, remodeling, and a healed appearance based on morphology and signal intensity. This educational framework is based on single-institution experience and clinical observation rather than a validated reinjury prediction model. Within it, mild residual swelling or fusiform connective-tissue thickening may be compatible with normal healing, whereas persistent intermuscular edema, a new connective-tissue tear, or scar rupture may represent atypical recovery (10).

For RTP decisions, the focus should not be whether the scan has normalized, but whether the imaging pattern is expected for the stage of healing and whether it adds information to clinical and functional findings. This framing reduces both over-reliance on residual signal abnormalities and under-recognition of potentially important structural findings.

## MRI abnormalities at RTP: common does not mean dangerous

Several reports, derived partly from overlapping Dutch and Qatari cohorts, show that residual MRI abnormalities are common at RTP. Reurink et al. found that 47 of 53 clinically recovered athletes (89%) still had increased intramuscular signal on fluid-sensitive sequences at RTP, and 42% had low-signal tissue (11). In a related pooled cohort, fibrosis was present at RTP in 41 of 108 athletes (38%); reinjury occurred in 24% of athletes both with and without fibrosis (HR 0.95, 95% CI 0.43–2.10) (12). These findings argue against requiring MRI normalization, but the publications should not be interpreted as independent replications because their source cohorts overlap.

A more recent 3.0-T MRI study similarly documented frequent edema, low-signal fibrotic change, intramuscular tendon disruption, waviness, and tendon thickening at clinical RTP (13). Only three reinjuries occurred among 58 participants, so the study was primarily descriptive and could not determine whether particular residual findings predict reinjury. Higher-resolution imaging may reveal more residual abnormalities, but detection alone does not establish clinical relevance.

Intratendinous and intramuscular tendon involvement is the area in which the evidence is most heterogeneous. The British Athletics Muscle Injury Classification (BAMIC), which uses the ‘c’ suffix for intratendinous injury, was proposed as an evidence-informed framework that required validation (14). In 44 elite track-and-field athletes with 65 hamstring injuries, including 31 sprinters and eight jumpers, Pollock et al. reported recurrence in 5 of 8 BAMIC grade 2c injuries and 4 of 7 grade 3c injuries, compared with 1 of 17 grade 2b injuries and none of the myofascial injuries; six recurrences occurred before return to full training; 14 athletes (32%) contributed more than one injury (15). In that retrospective series, injury grade itself was not associated with recurrence (*p* = 0.133–0.968), whereas intratendinous site was (*p* = 0.002) (15). A separate RTP-MRI cohort subsequently showed that tendon discontinuity can persist after clinical recovery (16). By contrast, van der Made et al. pooled 165 athletes, 72% of whom were footballers, and found identical 12-month reinjury proportions with and without baseline intramuscular tendon disruption (20% vs. 20%; HR 1.05, 95% CI 0.52–2.12) (17). *Post-hoc* analyses at 2 and 3 months after RTP pointed in the same direction as Pollock et al. but remained imprecise: adjusted HR 2.43 (95% CI 0.91–6.51; *p* = 0.077) at 2 months and 2.43 (95% CI 0.96–6.12; *p* = 0.061) at 3 months (17). The authors also cautioned that their predominantly team-sport results should not be generalized to track-and-field athletes (17). The tension between these studies may therefore reflect sport-specific sprint demands, outcome timing, cohort composition, and limited precision rather than a settled conclusion. Importantly, van der Made et al. (17) assessed MRI at baseline, not at RTP. At RTP, Vermeulen et al. found residual partial- or complete-thickness tendon discontinuity in 56% of 41 athletes; eight reinjuries occurred during follow-up, and tendon characteristics did not differ detectably between reinjured and non-reinjured athletes, but the small number of events cannot exclude a clinically meaningful association (16). Tendon findings should therefore inform phenotype- and sport-specific risk management, but neither baseline nor residual tendon abnormality is a validated stand-alone RTP criterion.

MRI signal intensity alone appears to have limited value as an RTP criterion. Van der Horst et al. found that STIR signal intensity at RTP, and changes in STIR signal over time, were not associated with reinjury risk (18). Paradoxically, greater STIR signal at the initial injury was weakly associated with a lower reinjury risk in one analysis (18), further emphasizing that edema-like signal is nonspecific and should not be interpreted as a binary clearance variable.

## Which MRI findings may be more concerning?

Some structural MRI patterns warrant closer evaluation, but the available prognostic evidence is exploratory. In a single-club pilot study of 59 professional athletes with lower-limb muscle injuries, including 40 hamstring injuries, Isern-Kebschull et al. recorded nine reinjuries within 2 months (19). On pre-RTP MRI, transversal and/or mixed connective-tissue gap (OR 39.2, 95% CI 3.64–422.14; *p* = 0.002), intermuscular edema (OR 8.17, 95% CI 1.52–43.99; *p* = 0.015), and callus gap (OR 5.75, 95% CI 1.03–32.17; *p* = 0.046) were associated with reinjury at the conventional *p* < 0.05 level, whereas loss of tendon tension (OR 2.63, 95% CI 0.54–12.7; *p* = 0.231) and interstitial feather edema (OR 1.87, 95% CI 0.45–7.85; *p* = 0.389) were not individually associated (19). The study specified *p* = 0.01 as its significance threshold, which only the connective-tissue gap met. A composite score of at least two of five candidate signs was associated with reinjury, but the small event count, wide confidence intervals, mixed muscle sample, short follow-up, and lack of external validation preclude treating the five signs as established RTP contraindications.

To make these five candidate signs reproducible, Isern-Kebschull et al. (19) operationalized them on pre-RTP MRI in comparison with baseline imaging. A transversal/mixed connective-tissue gap was a new or persistent measurable hyperintense gap in thick connective tissue on fluid-sensitive sequences, oriented transversely or both transversely and longitudinally. Loss of tendon tension was loss of normal taut longitudinal alignment, producing an undulating or wavy tendon on long-axis images. Intermuscular edema was fluid-sensitive hyperintensity or fluid between adjacent muscles, whereas interstitial feather edema was persistent or progressive feathery hyperintensity tracking along muscle fibers. A callus gap was the persistence or appearance of a hyperintense gap on T2-weighted images with clear transverse loss of continuity through the repair callus (19). These definitions depend on standardized fluid-sensitive planes and baseline comparison and should not be extrapolated directly to ultrasound.

Baseline MRI may still contribute to risk profiling. In a multicentre analysis of 330 acute hamstring injuries, Zein et al. found that myotendinous-junction involvement and less anteroposterior edema on baseline MRI, together with selected clinical variables and shorter time to RTP, were independently associated with 12-month reinjury (20). However, this analysis pooled four study registrations and overlaps with cohorts used in several earlier publications, so it should not be considered independent confirmation of those reports. The counterintuitive association with less edema also cautions against simple signal-based risk rules. Baseline MRI may therefore characterize injury phenotype and inform rehabilitation planning, but it does not itself clear RTP.

In practice, MRI interpretation near RTP should give greater weight to tissue architecture than to nonspecific signal intensity, while maintaining appropriate uncertainty. Residual edema, mature scar thickening, and tendon thickening may be expected during remodeling. Persistent or new connective-tissue gaps, intermuscular edema, callus gaps, or a new scar tear may justify closer multidisciplinary reassessment; however, current evidence does not validate any of these findings as an automatic reason to delay RTP. Loss of tendon tension should be regarded as an exploratory structural concern rather than an established predictor because its individual association with reinjury was null in that study (19).

## Ultrasound: pragmatic follow-up tool, not a complete substitute

In managing hamstring injuries, ultrasound has practical advantages. It can be performed rapidly, repeated during rehabilitation, used dynamically, and is less costly than MRI. In experienced hands, it can identify hematomas, structural disruption, tendon involvement, and evolving scar. These characteristics make ultrasound attractive for serial assessment when repeated MRI is impractical (5–7).

Ultrasound also has important limitations. It is less suited than MRI to some deep proximal injuries, complex intramuscular tendon lesions, subtle connective-tissue gaps, and multiplanar assessment, and image quality depends strongly on operator skill, probe selection, body habitus, and scanning technique. In an earlier longitudinal cohort, 10 of 45 injured players (22.2%) still had an abnormal sonogram at 6 weeks, although all but one player had returned to competition; MRI remained more sensitive for follow-up assessment (6). In a 2025 study of acute first-time hamstring strains, ultrasound and MRI agreed on injury location in 70% of cases overall, with agreement of 90% for muscle-belly injuries, 80% for musculotendinous-junction injuries, and only 60% for tendon injuries (7). The same study included 21 modified Peetrons grade 3 injuries: 20 musculotendinous-junction grade 3 injuries returned to sport in 100 ± 27 days versus 57 ± 21 days for grade 2 injuries at the same site, while the single grade 3 tendon injury was treated by surgical reattachment and returned at 383 days (7). The authors concluded that MRI remained important for detailed assessment of high-grade tendon lesions (7). These data support complementary use of the modalities, particularly because tendon morphology is one of the phenotypes of greatest concern in this review.

Evidence specifically linking ultrasound findings at RTP to subsequent reinjury is currently insufficient. Ultrasound may be useful for serial evaluation of a targeted structural question, such as persistence of a hematoma or an accessible architectural defect, but the cited literature does not establish an ultrasound threshold that defines safe loading or safe RTP. For complete proximal tendon avulsions, surgically treated lesions, or other high-grade presentations, the RTP studies synthesized here provide little direct evidence; such cases require separate tendon- and surgery-specific assessment rather than extrapolation from predominantly nonoperative cohorts.

## Integrating imaging into RTP decision-making

Return to play after hamstring injury should be criteria based rather than imaging based. Reviews and consensus statements support integrating clinical status, strength, function, psychological readiness, and sport-specific exposure (21–24). An earlier systematic review found no strong evidence that any single MRI feature robustly predicts time to RTP, although selected baseline findings may inform prognosis (25). Empirical data also support this hierarchy. In de Vos et al., none of the baseline MRI variables predicted reinjury, whereas previous hamstring injury, active knee-extension deficit, isometric knee-flexion force deficit, and localized discomfort on palpation measured just after RTP were independent predictors (26). Wangensteen et al. found that adding MRI variables to a model based on history and clinical examination increased the adjusted R-squared for time to return to sport only from 0.290 to 0.318, an additional 2.8% of explained variance (27). Professional-football data have linked selected baseline MRI characteristics with RTP duration (28). A systematic review of nonoperative hamstring management also identified multiple clinical and imaging determinants of RTP (29). Imaging is therefore best considered a context-dependent modifier whose value depends on whether it adds information beyond clinical and functional assessment.

The studies summarized here use different classification vocabularies. Modified Peetrons grading was explicitly used in several MRI cohorts (7, 11–13, 17, 20). Van der Horst et al. (18) used grade I–II eligibility inherited from the parent Dutch trial but did not name the grading system in that report; de Vos et al. (26) and Wangensteen et al. (27) also used MRI grade 1–2 eligibility, with grade 3 excluded in the latter, but are not treated here as evidence that the same named grading system was explicitly reported in those articles. BAMIC was introduced by Pollock et al. (14) and applied by Zein et al. (13) and Pollock et al. (15), whereas MLG-R was introduced by Valle et al. (30) and used by Isern-Kebschull et al. (19). Isern-Kebschull et al. (10) proposed a phase-based MRI healing framework rather than an injury-severity scale. These systems are not interchangeable. In Zein et al. (13), the same 58 baseline injuries were all modified Peetrons grade 1–2 (15 grade 1 and 43 grade 2), while BAMIC classified 30 of 58 (52%) as grade 3 (2 grade 3A, 18 grade 3B, and 10 grade 3C). Accordingly, numerical grades in this review are qualified by the relevant system, and no grade is mapped directly from one classification to another.

To translate the evidence cautiously, we propose a conceptual, non-validated three-tier framework. First, expected remodeling includes minor residual edema-like signal, mature low-signal scar/fibrosis, fusiform connective-tissue thickening, and tendon thickening without loss of continuity; these findings should not independently prevent RTP after clinical and functional criteria are met (10–13, 16, 18). Second, uncertain residuals include persistent intramuscular tendon discontinuity, peritendinous edema, minor scar heterogeneity, loss of tendon tension, and isolated persistent or progressive interstitial feather edema; the latter two were biologically plausible components of the five-sign set but were not individually associated with reinjury in the study by Isern-Kebschull et al. (19). Third, candidate structural warning signs include persistent or new connective-tissue gap, intermuscular edema, callus gap, and a new connective-tissue tear or scar rupture. The first three have only exploratory outcome data from a small single-club cohort (19), whereas scar rupture/new tear is supported primarily by the proposed healing framework (10) rather than independent reinjury validation. These findings should trigger multidisciplinary reassessment and may modify or delay RTP when concordant with symptoms, strength deficits, inadequate high-speed exposure, or other risk factors; they are not absolute imaging contraindications.

The RTP decision should therefore address five linked questions. Is the athlete pain free during maximal or near-maximal sprinting, kicking, and sport-specific movements? Has hamstring strength, particularly eccentric strength at long muscle lengths, recovered to an acceptable level? Has the athlete tolerated progressive high-speed running exposure without symptom recurrence? Does the injury phenotype or sport impose a higher mechanical reinjury risk, for example substantial myotendinous or intratendinous involvement in a sprint-dominant athlete? Finally, does imaging show expected remodeling, an uncertain residual finding, or a candidate structural concern that is concordant with the clinical and performance picture?

When ultrasound is used in this framework, the examination should address a pre-specified, accessible structural target and serial studies should be technically comparable. Without a defined target and standardized acquisition, ultrasound is unlikely to add reliable information to a criteria-based RTP decision and may create false reassurance or unnecessary delay (5–7).

**Figure 1:**
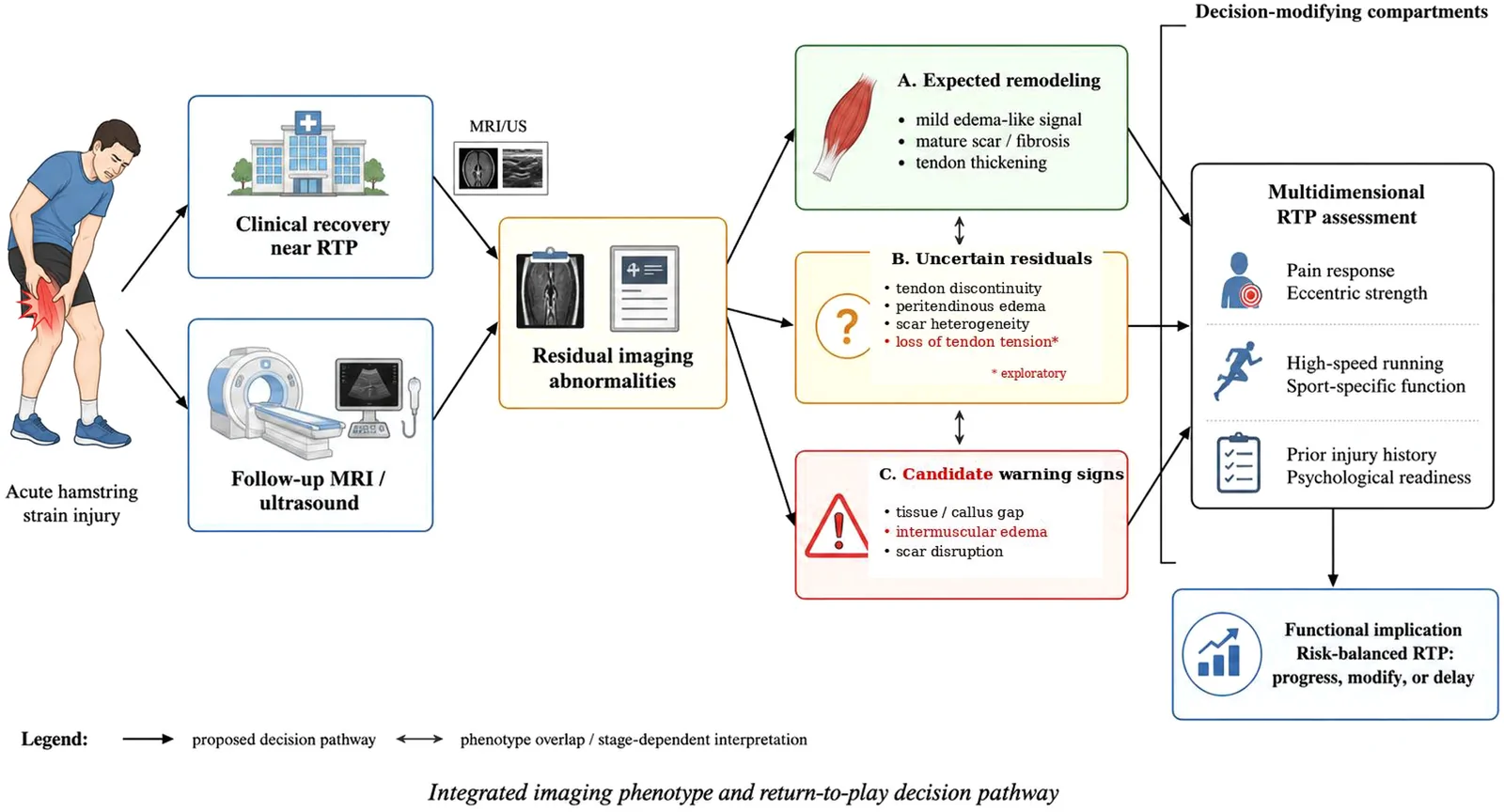
Proposed conceptual framework for using imaging in return-to-play decisions after acute hamstring strain injury. The framework separates residual findings into expected remodeling, uncertain residuals, and candidate warning signs. It is hypothesis-generating and has not been validated as an RTP algorithm. Imaging interpretation should be integrated with pain-free sport-specific exposure, eccentric strength, flexibility, high-speed running load, prior injury history, and psychological readiness. Candidate warning signs should trigger reassessment rather than automatic restriction; loss of tendon tension is exploratory and was not individually associated with reinjury in the study by Isern-Kebschull et al. (19). MRI or ultrasound should modify the RTP decision when structural findings are concerning or when clinical and functional findings are discordant. US, ultrasound.

This approach avoids two weak practices: clearing an athlete because the scan appears improved and delaying an athlete solely because the scan has not normalized. Table 1 summarizes the proposed interpretation of residual imaging findings near RTP, and Figure 1 presents a conceptual pathway for integrating imaging phenotype with multidimensional RTP assessment.

**Table 1:** Interpreting residual imaging abnormalities near RTP.

Imaging finding near RTP	Likely interpretation	Suggested clinical response	Evidence basis**/**strength
Mild residual intramuscular edema on fluid-sensitive MRI	Common remodeling feature; nonspecific	Do not delay RTP solely for this finding; confirm tolerance to high-speed running	Descriptive RTP MRI evidence (11, 13, 18); cohorts partly overlap. Low prognostic specificity.
Low-signal scar/fibrosis	Expected scar maturation in many athletes	Interpret with symptoms and function; fibrosis alone is not a proven reinjury marker	108-athlete related cohort: reinjury 24% with vs. 24% without fibrosis; HR 0.95 (12).
Tendon thickening without loss of continuity	Healing/remodeling of intramuscular tendon	Continue criteria-based progression; monitor sprint and eccentric-load response	Descriptive RTP tendon data (16) plus proposed healing framework (10); reinjury analysis underpowered.
Persistent intramuscular tendon discontinuity	Uncertain significance; may persist despite clinical success	Increase caution in phenotype- and sport-specific context; review strength, sprint exposure, and prior injury	56% at RTP in 41 athletes; 8 reinjuries (16). Limited event count; no definitive risk estimate.
Loss of tendon tension	Exploratory structural concern; not an established reinjury predictor	Interpret with the full clinical/functional picture; do not delay RTP solely for this finding	Individually null in the single-club pilot: OR 2.63 (95% CI 0.54–12.7), *p* = 0.231 (19). Exploratory.
Persistent/progressive interstitial feather edema	Uncertain residual when isolated; component of an exploratory composite sign set	Interpret with architecture, symptoms, and function; do not delay RTP solely for this finding	Individually null in the single-club pilot: OR 1.87 (95% CI 0.45–7.85), *p* = 0.389 (19). Exploratory.
Persistent intermuscular edema	Candidate structural concern, particularly late in recovery	Reassess symptoms, loading progression, and structural healing; do not use as a stand-alone delay criterion	Single-club pilot: OR 8.17, *p* = 0.015; did not meet the study's *p* = 0.01 threshold (19). Exploratory.
Transversal/mixed connective-tissue gap or callus gap	Possible abnormal structural healing	Multidisciplinary reassessment; may modify or delay RTP if concordant with clinical/functional concerns	Single-club pilot: tissue gap OR 39.2, *p* = 0.002; callus gap OR 5.75, *p* = 0.046 (19). Exploratory.
Scar rupture or new connective-tissue tear	Potential abnormal healing/new structural failure	Reassess diagnosis, rehabilitation load, symptoms, and mechanism before progression	Proposed healing framework (10); biologically plausible but not independently validated against hamstring reinjury.
Ultrasound-detected unresolved hematoma or architectural defect	Context-dependent structural finding	Use standardized serial assessment for a defined, accessible target; consider MRI for deep or tendon-related lesions; not a sole clearance test	Longitudinal follow-up evidence (6) and acute diagnostic/prognostic evidence (7); no cited ultrasound-at-RTP reinjury threshold.

## Discussion

Current evidence does not support requiring MRI or ultrasound normalization before RTP after an acute hamstring strain injury. Even in clinically recovered athletes, residual MRI abnormalities are frequent, and edema-like signal, fibrosis, and persistent tendon changes can coexist with successful RTP (11–13, 16, 18). This should discourage defensive delays based solely on incomplete imaging normalization, while recognizing that several of these studies arise from overlapping cohorts and have limited reinjury event counts.

Completely disregarding imaging is also overly simplistic. Structural abnormalities involving connective-tissue continuity may be more biologically plausible as concerns than nonspecific signal change, but the prognostic evidence is preliminary. The candidate warning-sign concept is derived mainly from a small single-club pilot study (19) and a proposed single-institution healing framework (10); neither provides independent validation of a stand-alone RTP rule. Accordingly, connective-tissue gaps, intermuscular edema, callus gaps, or new scar disruption should prompt contextual reassessment rather than automatic delay, and loss of tendon tension should remain an exploratory finding. The future role of imaging is therefore not routine normalization scanning, but improved phenotyping and demonstration of incremental value beyond clinical and functional assessment.

Ultrasound has a distinct decision role rather than serving as a lower-cost surrogate for MRI. Its greatest potential is targeted longitudinal assessment of an accessible superficial lesion—for example, hematoma resolution, restoration of fiber architecture, or continuity of a visible tendon—using the same patient position, probe, scanning plane, and operator where possible. The longitudinal study in which 22.2% of athletes still had an abnormal sonogram at 6 weeks despite all but one having returned to competition illustrates why persistence alone cannot define an RTP threshold (6). Conversely, lower ultrasound–MRI agreement for tendon injuries and limited access to deep proximal anatomy mean that a nondiagnostic or clinically discordant ultrasound should prompt MRI rather than reassurance (5–7). A normal or improved ultrasound therefore cannot independently clear RTP, while an abnormal study should modify the decision only when it answers a pre-specified structural question and is concordant with symptoms and function.

Several limitations of the evidence base require explicit consideration. First, many publications derive from a small number of overlapping Dutch and Qatari cohorts. Studies (11, 12) draw on the same two trials (16, 17); pool those same Dutch/Qatari sources (18); uses the Dutch trial alone (13); is a substudy of the NL55671.018.16 diffusion-tensor-imaging cohort; and (20) pools four registrations, including source cohorts underlying (13, 16, 17) and related earlier reports. These publications should therefore not be interpreted as independent replications. Studies (10, 19) arise from the same broader clinical group and have substantially overlapping authorship. Second, reinjury counts are often low, injury and RTP definitions vary, and imaging schedules differ. Third, the literature uses different classification systems—including modified Peetrons, the British Athletics Muscle Injury Classification (BAMIC), MLG-R, and the phase-based healing scheme in (10)—and their numerical grades or categories are not interchangeable (7, 10, 13–15, 19, 20, 30). BAMIC and MLG-R were proposed as evidence-informed/expert classifications that required validation (14, 30); MLG-R was also developed by a group with author overlap with the study in (19), reinforcing the need for external validation. The framework in (10) is a healing-phase scheme rather than an injury-severity grading system. Fourth, most prognostic cohorts using the modified Peetrons system were limited to grade 1–2 injuries and excluded complete free-tendon tears, proximal avulsions, or surgically treated cases, restricting generalizability to the most severe presentations. Finally, blinding differed by study: treating physiotherapists were blinded to MRI findings in the major Dutch/Qatari source cohorts (11, 12, 16–18), whereas pre-RTP MRI formed part of routine clinical management in the single-club studies (10, 19). Management-related bias is therefore relevant to some, but not all, of the literature.

Future studies should test integrated decision models in independent, sport-specific cohorts. The key question is not whether an MRI or ultrasound abnormality is present at RTP, but whether a defined modality-specific phenotype improves prediction beyond pain, flexibility, strength, high-speed running exposure, training load, previous injury, and psychological readiness. Existing work illustrates why this incremental approach matters: clinical findings near RTP predicted reinjury when baseline MRI variables did not (26), and MRI added only 2.8% explained variance to a history-and-examination model for time to return to sport (27). Future models should report discrimination, calibration, and clinical utility, include adequate reinjury event numbers, externally validate candidate structural signs, and deliberately include underrepresented high-grade and surgically managed phenotypes when clinically appropriate. Imaging variables also need precise definitions: the term edema is too broad unless its location, pattern, relationship to connective tissue, healing stage, and temporal evolution are specified. Ultrasound studies additionally require standardized patient positioning, transducer and scanning planes, operator training, explicit target lesions, intra- and interobserver reliability, serial time points, and direct testing of incremental value beyond clinical and functional assessment.

Quantitative MRI and ultrasound-based measures may eventually improve this process. In a small independent cohort of 20 collegiate athletes imaged at return to sport, diffusion-tensor-imaging measures were associated with eccentric strength, whereas no MRI-based measure was associated with reinjury; only four reinjuries occurred, so these findings remain preliminary (31). Ultrasound shear-wave elastography and machine-learning-based analysis of either modality remain investigational in this RTP context and must demonstrate that they improve decisions and outcomes rather than merely generate additional measurements. Clinically, the appropriate position remains selective: imaging should neither clear an athlete on its own nor prevent participation solely because an abnormality persists. It is most defensible when symptoms and function are discordant, structural injury is complex, recurrence has occurred, or the consequences of a small risk change are substantial. In lower-risk cases with clear clinical progression, routine RTP imaging is unlikely to be necessary; complete tendon avulsions and surgically managed injuries require a separate evidence framework.

## Conclusion

Current evidence does not establish that MRI or ultrasound can reliably distinguish incomplete healing from safe RTP when used in isolation. Residual MRI abnormalities after acute hamstring strain are common, and edema-like signal, mature fibrosis, and persistent tendon changes may be compatible with successful clinical recovery. Structural findings such as connective-tissue gaps or intermuscular edema may deserve greater concern, but their prognostic evidence remains exploratory, derives from small and partly overlapping cohorts, and is not sufficient to define absolute RTP contraindications. Evidence for ultrasound at RTP and for reinjury prediction is even more limited; ultrasound is best viewed as a pragmatic, targeted serial assessment tool, while MRI provides more detailed evaluation of deep and tendon-related anatomy. RTP decisions should therefore integrate imaging phenotype with pain, strength, flexibility, high-speed running exposure, sport-specific function, previous injury, and psychological readiness. The priority for future research is not more routine imaging, but independent prospective validation of whether specific imaging findings add clinically useful information beyond established clinical and functional criteria.

## References

[1] Ekstrand J Hägglund M Waldén M. Epidemiology of muscle injuries in professional football (soccer). Am J Sports Med. (2011) 39:1226–32. 10.1177/0363546510395879 21335353

[2] Erickson LN Sherry MA. Rehabilitation and return to sport after hamstring strain injury. J Sport Health Sci. (2017) 6:262–70. 10.1016/j.jshs.2017.04.001 30356646 PMC6189266

[3] Wangensteen A Tol JL Witvrouw E Van Linschoten R Almusa E Hamilton B. Hamstring reinjuries occur at the same location and early after return to sport: a descriptive study of MRI-confirmed reinjuries. Am J Sports Med. (2016) 44:2112–21. 10.1177/0363546516646086 27184543

[4] Heiderscheit BC Sherry MA Silder A Chumanov ES Thelen DG. Hamstring strain injuries: recommendations for diagnosis, rehabilitation, and injury prevention. J Orthop Sports Phys Ther. (2010) 40:67–81. 10.2519/jospt.2010.3047 20118524 PMC2867336

[5] Koulouris G Connell D. Imaging of hamstring injuries: therapeutic implications. Eur Radiol. (2006) 16:1478–87. 10.1007/s00330-005-0075-3 16514470

[6] Connell DA Schneider-Kolsky ME Hoving JL Malara F Buchbinder R Koulouris G. Longitudinal study comparing sonographic and MRI assessments of acute and healing hamstring injuries. AJR Am J Roentgenol. (2004) 183:975–84. 10.2214/ajr.183.4.1830975 15385289

[7] Hirahata Y Yasui Y Sasahara J Inui T Nakagawa T Kawano H. Role of ultrasonography and MRI in acute hamstring strains: diagnostic and prognostic insights. Diagnostics. (2025) 15:1053. 10.3390/diagnostics15091053 40361871 PMC12071714

[8] Järvinen TAH Järvinen TLN Kääriäinen M Kalimo H Järvinen M. Muscle injuries: biology and treatment. Am J Sports Med. (2005) 33:745–64. 10.1177/0363546505274714 15851777

[9] Mackey AL Kjaer M. The breaking and making of healthy adult human skeletal muscle *in vivo*. Skelet Muscle. (2017) 7:24. 10.1186/s13395-017-0142-x 29115986 PMC5688812

[10] Isern-Kebschull J Mechó S Pedret C Pruna R Alomar X Kassarjian A. Muscle healing in sports injuries: MRI findings and proposed classification based on a single institutional experience and clinical observation. Radiographics. (2024) 44:e230147. 10.1148/rg.230147 39052498

[11] Reurink G Goudswaard GJ Tol JL Almusa E Moen MH Weir A. MRI Observations at return to play of clinically recovered hamstring injuries. Br J Sports Med. (2014) 48:1370–6. 10.1136/bjsports-2013-092450 24255767 PMC4174122

[12] Reurink G Almusa E Goudswaard GJ Tol JL Hamilton B Moen MH. No association between fibrosis on magnetic resonance imaging at return to play and hamstring reinjury risk. Am J Sports Med. (2015) 43:1228–34. 10.1177/0363546515572603 25748473

[13] Zein MI Reurink G Suskens JJM Monte JRC Smithuis FF Buckens S. 3.0-Tesla MRI observation at return to play after hamstring injuries. Clin J Sport Med. (2025) 35:119–26. 10.1097/JSM.0000000000001289 39565178 PMC11837960

[14] Pollock N James SLJ Lee JC Chakraverty R. British Athletics muscle injury classification: a new grading system. Br J Sports Med. (2014) 48:1347–51. 10.1136/bjsports-2013-093302 25031367

[15] Pollock N Patel A Chakraverty J Suokas A James SLJ Chakraverty R. Time to return to full training is delayed and recurrence rate is higher in intratendinous (‘c’) acute hamstring injury in elite track and field athletes: clinical application of the British athletics muscle injury classification. Br J Sports Med. (2016) 50:305–10. 10.1136/bjsports-2015-094657 26888072

[16] Vermeulen R Almusa E Buckens S Six W Whiteley R Reurink G. Complete resolution of a hamstring intramuscular tendon injury on MRI is not necessary for a clinically successful return to play. Br J Sports Med. (2021) 55:397–402. 10.1136/bjsports-2019-101808 32561516

[17] van Der Made AD Almusa E Reurink G Whiteley R Weir A Hamilton B. Intramuscular tendon injury is not associated with an increased hamstring reinjury rate within 12 months after return to play. Br J Sports Med. (2018) 52:1261–6. 10.1136/bjsports-2017-098725 29654058

[18] van Der Horst RA Tol JL Weir A Den Harder JM Moen MH Maas M. The value of MRI STIR signal intensity on return to play prognosis and reinjury risk estimation in athletes with acute hamstring injuries. J Sci Med Sport. (2021) 24:855–61. 10.1016/j.jsams.2021.02.008 33622615

[19] Isern-Kebschull J Pedret C Mechó S Pruna R Alomar X Yanguas X. MRI Findings prior to return to play as predictors of reinjury in professional athletes: a novel decision-making tool. Insights Imaging. (2022) 13:203. 10.1186/s13244-022-01341-1 36575363 PMC9794673

[20] Zein MI Mokkenstorm MJK Cardinale M Holtzhausen L Whiteley R Moen MH. Baseline clinical and MRI risk factors for hamstring reinjury showing the value of performing baseline MRI and delaying return to play: a multicentre, prospective cohort of 330 acute hamstring injuries. Br J Sports Med. (2024) 58:766–76. 10.1136/bjsports-2023-107878 38729628 PMC11228232

[21] Ardern CL Glasgow P Schneiders A Witvrouw E Clarsen B Cools A. 2016 Consensus statement on return to sport from the first world congress in sports physical therapy, Bern. Br J Sports Med. (2016) 50:853–64. 10.1136/bjsports-2016-096278 27226389

[22] van der Horst N Backx FJG Goedhart EA Huisstede BMA; HIPS-Delphi Group. Return to play after hamstring injuries in football (soccer): a worldwide Delphi procedure regarding definition, medical criteria and decision-making. Br J Sports Med. (2017) 51:1583–91. 10.1136/bjsports-2016-097206 28360143

[23] Perna P Kerin F Greig N Beato M. Return-to-play criteria following a hamstring injury in professional football: a scoping review. Res Sports Med. (2025) 33:175–94. 10.1080/15438627.2024.2439274 39666593

[24] Pecci J Van Dyk N Myer GD Sañudo B. Return-to-play criteria following lower limb muscle injuries in soccer: a systematic review with evidence synthesis. Sports Med. (2026) 56:1433–65. 10.1007/s40279-026-02404-9 41851593 PMC13260053

[25] Reurink G Brilman EG De Vos R-J Maas M Moen MH Weir A. Magnetic resonance imaging in acute hamstring injury: can we provide a return to play prognosis? Sports Med. (2015) 45:133–46. 10.1007/s40279-014-0243-1 25119158

[26] De Vos RJ Reurink G Goudswaard GJ Moen MH Weir A Tol JL. Clinical findings just after return to play predict hamstring re-injury, but baseline MRI findings do not. Br J Sports Med. (2014) 48:1377–84. 10.1136/bjsports-2014-093737 25037201

[27] Wangensteen A Almusa E Boukarroum S Farooq A Hamilton B Whiteley R. MRI Does not add value over and above patient history and clinical examination in predicting time to return to sport after acute hamstring injuries: a prospective cohort of 180 male athletes. Br J Sports Med. (2015) 49:1579–87. 10.1136/bjsports-2015-094892 26305004

[28] Ekstrand J Healy JC Waldén M Lee JC English B Hägglund M. Hamstring muscle injuries in professional football: the correlation of MRI findings with return to play. Br J Sports Med. (2012) 46:112–7. 10.1136/bjsports-2011-090155 22144005

[29] Fournier-Farley C Lamontagne M Gendron P Gagnon DH. Determinants of return to play after the nonoperative management of hamstring injuries in athletes: a systematic review. Am J Sports Med. (2016) 44:2166–72. 10.1177/0363546515617472 26672025

[30] Valle X Alentorn-Geli E Tol JL Hamilton B Garrett WE Pruna R. Muscle injuries in sports: a new evidence-informed and expert consensus-based classification with clinical application. Sports Med. (2017) 47:1241–53. 10.1007/s40279-016-0647-1 27878524

[31] Wille CM Hurley SA Joachim MR Lee K Kijowski R Heiderscheit BC. Relationships between quantitative magnetic resonance imaging measures at the time of return to sport and clinical outcomes following acute hamstring strain injury. J Biomech. (2024) 173:112228. 10.1016/j.jbiomech.2024.112228 39032225 PMC11330723

